# D-galactose-induced mitochondrial DNA oxidative damage in the auditory cortex of rats

**DOI:** 10.3892/mmr.2014.2653

**Published:** 2014-10-15

**Authors:** ZHENGDE DU, QIONG YANG, TAO ZHOU, LIN LIU, SHUO LI, SHIXIONG CHEN, CHUNSHENG GAO

**Affiliations:** 1 Department of Otorhinolaryngology, Nanshan Affiliated Hospital of Guangdong Medical College, Shenzhen, Guangdong 518052, P.R. China; 2 The Chinese University of Hong Kong-Shenzhen Nanshan Hospital Joint Otorhinolaryngology, Head and Neck Surgery Training Centre cum Microsurgical and Endoscopic Skills Laboratory, Shenzhen, Guangdong 518052, P.R. China; 3 Department of Otorhinolaryngology, Union Hospital, Tongji Medical College, Huazhong University of Science and Technology, Wuhan, Hubei 430022, P.R. China; 4 College of Pharmacy, Guangxi Medical University, Nanning, Guangxi Zhuang Autonomous Region 530021, P.R. China; 5 Shenzhen Institutes of Advanced Technology, Chinese Academy of Sciences, Shenzhen, Guangdong 518055, P.R. China

**Keywords:** D-galactose, auditory cortex, oxidative damage, mitochondria, age-related hearing loss

## Abstract

Chronic administration of D-galactose (D-gal) is a useful method for establishing a model of natural aging in the auditory system. Previous studies have demonstrated that NADPH oxidases (NOXs) may be an important source of reactive oxygen species (ROS) in the peripheral auditory system (PAS) and cause an increase in mitochondrial DNA (mtDNA) common deletion (CD) levels in the PAS and central auditory system (CAS) of rats with D-gal-induced aging. However, the source of the ROS in the CAS and the mechanisms of age-related hearing loss (ARHL) have yet to be elucidated. In the present study, male Sprague Dawley rats were administered a daily injection of D-gal (150, 300 and 500 mg/kg, respectively) for eight weeks. All three doses of D-gal caused a significant increase in the expression of NOX2, 8-hydroxy-2-deoxyguanosine, a biomarker of DNA oxidative damage, and uncoupling protein 2, together with a decrease in the mitochondrial total antioxidant capabilities in the auditory cortex, as compared with the control rats (injected daily with the same volume of 0.9% saline for eight weeks). The levels of the mtDNA CD were also increased in the auditory cortex of the D-gal-induced aging rats. These findings suggest that both NOX- and mitochondria-associated ROS generation may contribute to mtDNA oxidative damage in the auditory cortex of the CAS of D-gal-induced aging rats. This study may provide novel insight into the development of ARHL.

## Introduction

Aging is the result of complex changes in the structure and function of molecules, cells, tissues and whole body systems. Age-related hearing loss (ARHL), also known as presbycusis, is believed to result from age-related degeneration of the peripheral and central components of the auditory system (1,2). Investigations into ARHL in humans is limited due to the inaccesibility of auditory system tissue and the complexity of the genetic and environmental background of individuals with hearing loss. Numerous animal models have been established in order to facilitate research into the molecular mechanisms of ARHL. Among them, an animal model using the chronic administration of D-galactose (D-gal) is widely used for studying the mechanisms of ARHL (3–11). These animals exhibit increased oxidative stress and mitochondrial DNA (mtDNA) common deletion (CD) in the peripheral and central auditory system (PAS and CAS, respectively); however, the source of the causative reactive oxygen species (ROS) in the CAS has not been fully investigated.

NADPH oxidases (NOXs) are one of the main ROS-generating sites. It is now clear that NOXs are not restricted to the immune system and that alternative isoforms may be active in numerous cell types as essential components of cellular signalling, gene expression regulation and cell differentiation. These enzymes are able to transport electrons across the plasma membrane, generating superoxide and other downstream ROS (12). The expression of NOX3 is higher in the PAS than that in any other tissue (13). In a previous study (5), it was demonstrated that NOX3 may be an important source of ROS in the PAS of rats with D-gal-induced aging and that chronic injection of D-gal could increase NOX3-dependent oxidative stress, mitochondrial damage and apoptosis in the PAS. NOX2 is not restricted to phagocytic cells, but is present in numerous non-phagocytic cells and tissues, including neurons of the CAS (14,15). The effects of NOX2 in the auditory cortex of the CAS of rats with D-gal-induced aging have yet to be elucidated.

Mitochondria are another site of predominant ROS generation in cells (16). Mitochondrial ROS generation is sensitive to the proton-motive force across the mitochondrial inner membrane produced by the electron transport chain, and mild uncoupling caused by the activation of uncoupling protein 2 (UCP2) may cause a reduction in the proton-motive force, attenuate mitochondrial ROS generation and protect cells against ROS-related cellular damage (17). It is therefore hypothesised that an overexpression of UCP2 may indirectly increase mitochondrial ROS generation. The expression of UCP2 in the CAS of rats with D-gal-induced aging, however, is unclear.

In the present study, the expression of NOX2, UCP2 and 8-hydroxy-2-deoxyguanosine (8-OHdG), a biomarker of DNA oxidative damage (18,19), as well as the mitochondrial total antioxidant capabilities (T-AOCs) and the levels of the mtDNA CD, were investigated in the auditory cortex of the CAS in a rat model with D-gal-induced aging. It was hypothesised that NOX- and mitochondria-dependent ROS generation and mtDNA oxidative damage may be primary etiological events in the degeneration of the CAS of rats with D-gal-induced aging.

## Material and methods

### Animals and treatments

Eighty-eight one-month-old male Sprague Dawley rats were obtained from the Experimental Animal Centre of Guangxi Medical University (Nanning, China). The rats were individually housed in temperature-controlled (20–22°C) conditions with a 12-h light/dark cycle, with free access to food and water. The body weight of each of the rats was monitored throughout the experiment as an indicator of health. Injections of D-gal to induce aging were administered according to established methodology (9). Following acclimation for two weeks, the rats were randomly divided into four groups (n=22 for each group), in which they were administered daily doses of D-gal (Sigma, St. Louis, MO, USA) or saline by subcutaneous injection for eight weeks. The three D-gal groups were referred to as the low- (150 mg/kg), medium- (300 mg/kg) and high- (500 mg/kg) dose groups and the fourth group was a control group (administered 0.9% saline at the same volume). At the end of the eight-week protocol, the rats were sacrificed by a terminal intraperitoneal injection of ketamine (30 mg/kg) and an intramuscular injection of chloropromazine (15 mg/kg). The auditory cortex was dissected and used for total RNA, genomic DNA and protein extraction and the examination of mitochondrial T-AOCs. Alternatively, the rats were perfused with 4% paraformaldehyde for immunohistochemical analysis. All experiments were carried out in strict accordance with the recommendations in the Guide for the Care and Use of Laboratory Animals of the National Institutes of Health. The protocol was approved by the Committee on the Ethics of Animal Experiments of Guangxi Medical University.

### RNA preparation and SYBR^®^ Green quantitative polymerase chain reaction (qPCR)

The mRNA expression levels of NOX2 and UCP2 were determined using a SYBR Green qPCR assay (Invitrogen Life Technologies, Carlsbad, CA, USA). Following the final injection of D-Gal, 24 rats (n=6 per group) were sacrificed, and both sides of the auditory cortex from each rat were rapidly removed. One side of the auditory cortex was used for RNA extraction, and the other side was used for mtDNA analysis. Total RNA was extracted with TRIzol^®^ reagent (Takara, Dalian, China) according to the manufacturer’s instructions. cDNA was reverse transcribed using a PrimeScript RT Reagent kit (Takara). The RNA and cDNA of each sample were analysed using a GeneQuant Pro DNA/RNA Calculator (Biochrom, Cambridge, UK) to assess the concentrations and purity. The cDNA samples were stored at −20°C until required. qPCR was performed by applying the SYBR Green qPCR technology with the use of a StepOnePlus™ Real-Time PCR system (Applied Biosystems, Foster City, CA, USA). The primer pairs for NOX2, UCP2 and β-actin (as an internal standard) were as follows: NOX2 forward, 5′-ACATTTTCGTCAAGCGTCCC-3′ and NOX2 reverse, 5′-CCCAGCTCCCACTAACATCA-3′; UCP2 forward, 5′-TGCTGGGCACCATCCTAACC-3′ and UCP2 reverse, 5′-CCTGGAAGCGGACCTTTACC-3′; β-actin forward, 5′-CCTGGAGAAGAGCTATGAGC-3′ and β-actin reverse, 5′-ACAGGATTCCATACCCAGG-3′. The amplification conditions were as follows: 30 sec at 95°C, then 40 cycles of 5 sec at 95°C, 30 sec at 60°C and 30 sec at 72°C. The amplification of β-actin as an internal standard was used to normalise the relative gene expression levels. A melting curve analysis was performed for each gene, and the specificity and integrity of the PCR products were confirmed by the presence of a single peak. The relative expression levels were calculated from the differences in the cycle threshold (Ct) values between the target mRNA and β-actin. The change in the relative mRNA levels between the experimental group and the control group was analysed using the 2^−ΔΔ^Ct method, as previously reported (20).

### Immunohistochemical analysis

The protein levels of NOX2 and the expression of 8-OHdG were determined by immunohistochemistry. Sixteen rats (n=4 per group) were sacrificed, and the brains were removed and fixed with 4% buffered paraformaldehyde overnight, dehydrated and embedded in paraffin wax. Serial sections of the brainstem were subsequently cut at a thickness of 5 μm at the level of the auditory cortex. The sections were then deparaffinised in xylene and rehydrated through graded concentrations of ethanol. The samples were incubated with anti-NOX2 (diluted 1:200; Boster Biological Technology Co., Ltd., Wuhan, China) and anti-8-OHdG (diluted 1:4,000; Abcam, Cambridge, MA, USA) antibodies overnight at 4°C. Following washes in phosphate-buffered saline, the slides were incubated with fluorescein isothiocyanate-conjugated anti-rabbit and Cy3-conjugated anti-mouse secondary antibodies (diluted 1:200; Boster Biological Technology Co., Ltd.) for 30 min at room temperature and the nuclei were counterstained using DAPI staining solution (Beyotime Institute of Biotechnology, Haimen, China) for 5 min at room temperature. Images were captured using a laser scanning confocal microscope (Nikon, Tokyo, Japan) and analysed using Image-Pro Plus 6.0 software (Media Cybernetics, Inc., Rockville, MD, USA). As a negative control, sections were treated in the same manner but without the incubation with primary antibody.

### Western blot analysis

The protein levels of UCP2 in the auditory cortex were determined using western blot analysis. Twenty-four rats (n=6 per group) were sacrificed, and both sides of the auditory cortex from each rat were dissected. The total protein was extracted using radioimmunoprecipitation assay buffer (Beyotime Institute of Biotechnology) according to the manufacturer’s instructions. Protein concentrations were determined using an Enhanced Bicinchoninic Acid Protein Assay kit (Beyotime Institute of Biotechnology). Protein lysate (30 μg) was separated by 12% SDS-PAGE and transferred to polyvinylidene difluoride membranes. The membranes were incubated for 1 h in a blocking solution [Tris-buffered saline (TBS) containing 5% skimmed milk], then washed briefly in TBS and incubated overnight at 4°C with the appropriate dilution of primary antibodies: Anti-UCP2 (diluted 1:500; Abcam) or anti-β-actin (diluted 1:1,000; Bioworld Technology, Inc., Minneapolis, MN, USA). The membranes were then washed to remove excess primary antibody and incubated for 1 h at room temperature with the appropriate horseradish peroxidase-conjugated secondary antibody (diluted 1:5,000; Santa Cruz Biotechnology, Inc., Santa Cruz, CA, USA). The membranes were visualised by the enhanced chemiluminescence method using BeyoECL Plus (Beyotime Institute of Biotechnology) reagent. A quantification of the detected bands was performed using Image-Pro Plus 6.0 software. β-actin was used as an internal control.

### Mitochondrial T-AOC determination

Twenty-four rats (n=6 per group) were sacrificed, and both sides of the auditory cortex from each rat were dissected. Mitochondria in the auditory cortex were quickly extracted using a Tissue Mitochondria Isolation kit (Beyotime Institute of Biotechnology) and subsequently used for the analysis of the T-AOCs. Mitochondrial T-AOCs in the auditory cortex were detected using a Total Antioxidant Capacity Assay kit in combination with the fluorescence recovery after photobleaching method, according to the manufacturer’s instructions (Beyotime Institute of Biotechnology).

### DNA isolation and TaqMan^®^ qPCR

Total DNA was extracted using the Genomic DNA Purification kit (Tiangen Biotech Co., Ltd, Beijing, China) according to the manufacturer’s instructions. The DNA concentration of each specimen was measured using the GeneQuant Pro DNA/RNA Calculator and the mtDNA CD levels were determined using a TaqMan qPCR assay. The displacement (D)-loop in the non-coding region of mtDNA, representing the conserved segment of mtDNA, was used as a measure of copy number. Primers and probes for the mtDNA D-loop and the mtDNA CD were designed as previously described (21). The PCR amplification was performed on a StepOnePlus Real-Time PCR system in a 20-μl reaction volume consisting of 10 μl 2× TaqMan PCR mix (Takara), 0.4 μl 50× ROX™ reference dye, 0.4 μl each forward and reverse primer (10 μM), 0.2 μl each probe (10 μM), 4 μl sample DNA (10 ng/μl) and 4.6 μl distilled water. The cycling conditions consisted of an initial phase at 95°C for 30 sec, then 40 cycles at 95°C for 5 sec and at 60°C for 30 sec. The cycle number at which a significant increase in the normalised fluorescence was first detected was designated as the Ct value. The ratio of the mtDNA CD to the mtDNA was calculated by the formula ΔCt=(Ct_mtDNA deletion_ - Ct_mtDNA D-loop_). The relative expression (RE) was used to indicate the factorial difference in the deletions between the experimental and control groups. The RE was calculated according to the 2^−ΔΔ^Ct method, where ΔΔCt = ΔCt_mtDNA deletion in experimental group_ - ΔCt_mtDNA deletion in control group_.

### Statistical analysis

The data are presented as the mean ± standard deviation. The analysis was performed using SPSS 13.0 software (SPSS, Inc., Chicago, IL, USA). Statistical significance was tested by a one-way analysis of variance. The least significant difference post hoc test was used to evaluate the differences between groups. P<0.05 was considered to indicate a statistically significant difference.

## Results

### D-gal induces an increase in NOX2 and UCP2 mRNA levels

**Figure 1:**
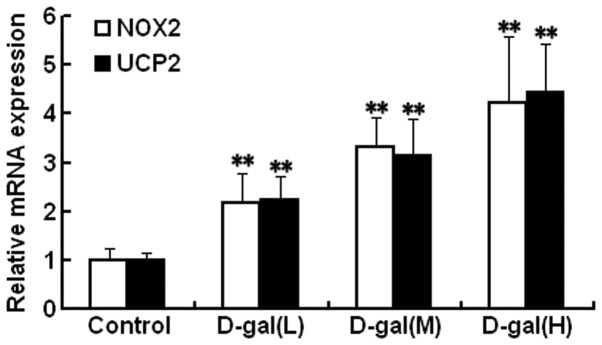
Quantitative analysis of NOX2 and UCP2 mRNA expression in the auditory cortex of rats in the different groups. The mRNA levels of NOX2 and UCP2 were significantly increased in the D-gal-treated rats as compared with those in the control rats. Data are expressed as the mean ± standard deviation of six rats per group. ^**^P<0.01 versus the control group. D-gal (L), low-dose D-gal group; D-gal (M), medium-dose D-gal group; D-gal (H), high-dose D-gal group; NOX2, NADPH oxidase; UCP2, mitochondrial uncoupling protein 2; D-gal, D-galactose.

To investigate the mRNA levels of NOX2 and UCP2 in the auditory cortex, SYBR Green qPCR was performed. As shown in Fig. 1, the mRNA levels of NOX2 and UCP2 were significantly higher in the D-gal-treated groups as compared with those in the control group (P<0.01).

### D-gal induces an increase in NOX2 and 8-OHdG protein expression

**Figure 2:**
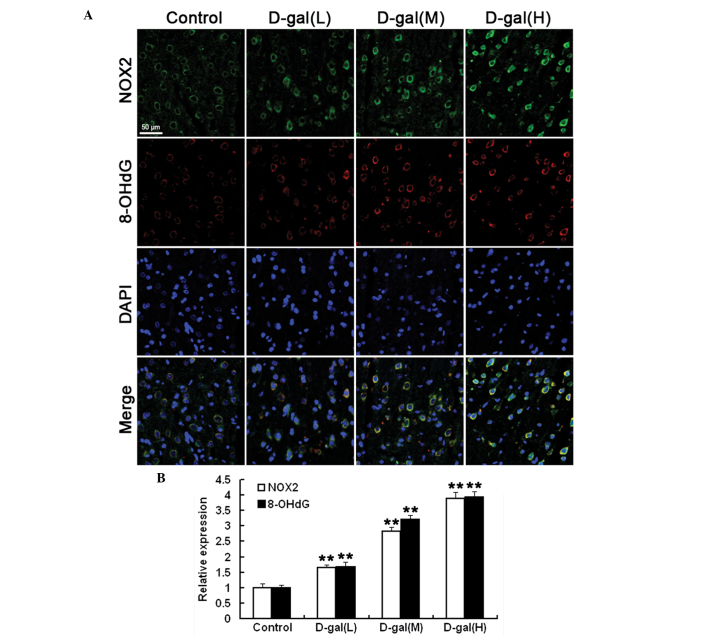
Protein levels of NOX2 and 8-OHdG expression in the auditory cortex of rats in the different groups. (A) Triple staining of rat brain sections with anti-NOX2 (green) and anti-8-OHdG (red) antibodies and DAPI for the staining of cellular nuclei (blue). The expression of 8-OHdG was predominantly localised to the cytoplasm of the cells. Scale bar, 50 μm; magnification, ×600. (B) Quantitative assessment of NOX2 and 8-OHdG expression in the auditory cortex. NOX2 and 8-OHdG expression in the auditory cortex was significantly increased in the D-gal-treated rats as compared with that in the control rats. Data are expressed as the mean ± standard deviation of four rats per group. ^**^P<0.01 versus the control group. D-gal (L), low-dose D-gal group; D-gal (M), medium-dose D-gal group; D-gal (H), high-dose D-gal group; NOX2, NADPH oxidase; 8-OHdG, 8-oxo-2′-deoxyguanosine; D-gal, D-galactose.

Immunohistochemical analysis was performed in order to investigate the protein levels of NOX2 and 8-OHdG expression in the auditory cortex. As shown in Fig. 2A and B, the NOX2 and 8-OHdG expression in the auditory cortex was significantly increased in the D-gal-treated groups as compared with that in the control group. Furthermore, the expression of 8-OHdG was predominantly localised to the cytoplasm of the cells in the auditory cortex (Fig. 2A) (P<0.01).

### D-gal induces an increase in UCP2 protein expression

**Figure 3:**
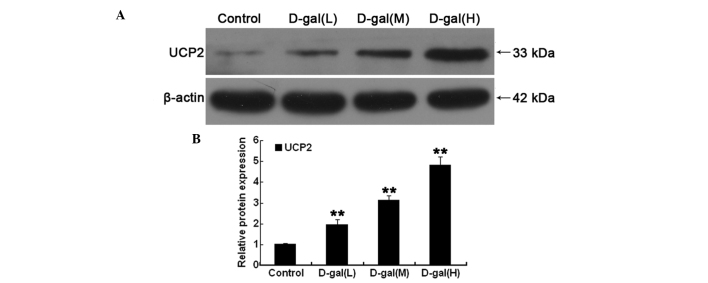
Western blot and densitometric analysis of UCP2 in the auditory cortex of rats in the different groups. (A) Representative western blots showing the expression levels of UCP2 in the different groups. (B) The relative abundance of UCP2 protein was significantly increased in the D-gal-treated groups compared with that in the control group. Data are expressed as the mean ± standard deviation of six rats per group. ^**^P<0.01 versus the control group. D-gal (L), low-dose D-gal group; D-gal (M), medium-dose D-gal group; D-gal (H), high-dose D-gal group; UCP2, mitochondrial uncoupling protein 2; kDa, kilodaltons; D-gal, D-galactose.

Western blot analysis was performed in order to investigate the protein levels of UCP2 in the auditory cortex. As shown in Fig. 3A and B, the protein levels of UCP2 were significantly higher in the D-gal-treated groups as compared with those in the control group (P<0.01).

### D-gal induces a decrease in mitochondrial T-AOC

**Figure 4:**
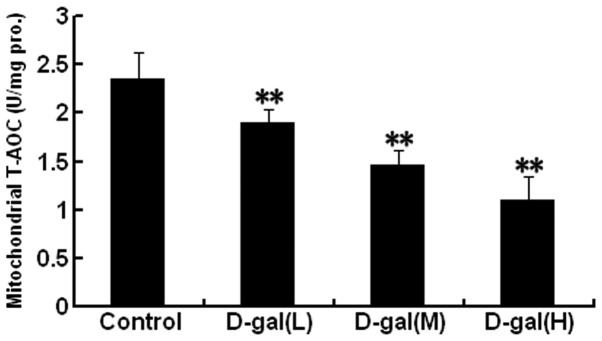
Levels of mitochondrial T-AOC in the auditory cortex of rats in the different groups. The mitochondrial T-AOC levels were significantly decreased in the D-gal-treated groups as compared with those in the control group. Data are expressed as the mean ± standard deviation of six rats per group. ^**^P<0.01 versus the control group. D-gal (L), low-dose D-gal group; D-gal (M), medium-dose D-gal group; D-gal (H), high-dose D-gal group; T-AOC, total antioxidant capability, D-gal, D-galactose.

Mitochondrial T-AOC was analysed in the auditory cortex. As shown in Fig. 4, the mitochondrial T-AOCs were significantly decreased in the D-gal-treated groups as compared with those in the control group (P<0.01).

### D-gal induces increased levels of the mtDNA CD

**Figure 5:**
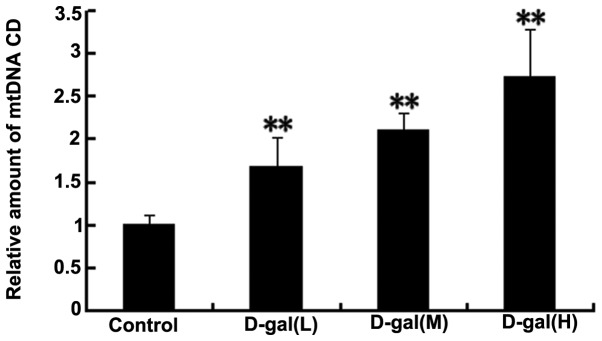
Quantitative analysis of the accumulation of the mtDNA CD in the auditory cortex of rats in the different groups. The levels of the mtDNA CD were significantly increased in the D-gal-treated groups as compared with those in the control group. Data are expressed as the mean ± standard deviation of six rats per group. ^**^P<0.01 versus the control group. mtDNA, mitochondrial DNA; CD, common deletion; D-gal (L), low-dose D-gal group; D-gal (M), medium-dose D-gal group; D-gal (H), high-dose D-gal group; D-gal, D-galactose.

TaqMan qPCR assay was used to investigate the levels of the mtDNA CD in the auditory cortex. The dual-labelled fluorescent DNA probe was designed to specifically recognise the fusion sequence, which was present only in mutant mtDNA that harboured the CD. As shown in Fig. 5, the accumulation of the mtDNA CD was significantly higher in the D-gal-treated groups as compared with that in the control group (P<0.01).

## Discussion

Oxidative damage to mtDNA has a strong association with the molecular process of aging (22). To the best of our knowledge, this study showed for the first time that levels of NOX2 and 8-OHdG, a biomarker of DNA oxidative damage, were significantly increased in the auditory cortex of the CAS in rats with D-gal-induced aging. The expression of 8-OHdG was observed predominantly in the cytoplasm of cells, suggesting that D-gal induced mtDNA oxidative damage in the auditory cortex. These findings indicate that D-gal may induce mtDNA oxidative damage in the CAS in part through the NOX2 pathway, and NOX2-associated ROS generation may play an essential role in the aging process of the CAS. NOXs function to produce ROS, as opposed to mitochondria, which generate ROS as a byproduct of their metabolism (23). Previous studies have demonstrated that NOX3 represents the primary source of ROS generation in the inner ear, which contributes to cisplatin-induced ROS generation in the PAS (13,24,25). It has additionally been shown that NOX3-associated ROS generation may function in the degeneration in the PAS (5), and NOX2-dependent oxidative stress may contribute to the mtDNA CD and mitochondrial ultrastructural damage in the hippocampus (26) of rats with D-gal-induced aging. Therefore, NOX2-associated ROS generation may be an important source of ROS in the aging process of the CAS.

Mitochondria are another important source of ROS in cells (16). UCP2, which is located in the mitochondrial inner membrane, can act to slightly lower the proton-motive force across the membrane through a mild uncoupling and, in this way, attenuate mitochondrial ROS generation (17). In a previous study, it was demonstrated that UCP2 mRNA expression was upregulated in the inner ear ganglia cells of the mouse following aminoglycoside intoxication, and these responses were blocked by a co-administration with antioxidant (27). Previous studies have also reported that UCP2 mRNA expression in the vestibular ganglion of the inner ear was significantly upregulated following a labyrinthectomy (28), and UCP2 mRNA and protein expression was significantly increased in the inner ear of D-gal- and/or high-fat diet-treated rats (5). These previous studies have indicated that UCP2 may indirectly reflect the ROS levels of cells and play a protective role against oxidative damage in the inner ear. In the present study, UCP2 was found to be overexpressed in the auditory cortex of rats with D-gal-induced aging, which may also suggest that increased UCP2 levels reflect increases in ROS generation and that UCP2 plays protective roles in the aging process of the CAS in rats with D-gal-induced aging.

mtDNA is highly susceptible to ROS-induced damage due to its close proximity to the sites of ROS generation and lack of protective histones (29). Increases in ROS generation together with decreases in mitochondrial T-AOCs may induce mtDNA oxidative damage. The most common type of mtDNA damage associated with aging is the mtDNA 4,977-bp deletion (also known as the CD) in humans and the corresponding mtDNA 4,834-bp deletion in rats. Therefore, the mtDNA CD has been widely used as a biomarker for aging (21,30–32). An association between elevated mtDNA CD levels and ARHL has been observed in a number of studies (32–34). Previous studies have demonstrated that the frequency of the mtDNA CD was increased in the PAS (5,6,8,10,11) and CAS (4,7,9) of D-gal-induced aging rats. The present study also found that the mtDNA CD levels were increased in the auditory cortex of D-gal-treated rats, and these increased mtDNA CD levels correlated with mtDNA oxidative damage. Therefore, these findings suggest that mtDNA damage in the auditory cortex of rats with D-gal-induced aging may be caused by NOX2- and mitochondria-associated ROS generation.

In conclusion, the present findings indicate that both NOX- and mitochondria-associated ROS generation may contribute to mtDNA oxidative damage in the auditory cortex of the CAS of rats with D-gal-induced aging. NOX2 and UCP2 may therefore be useful therapeutic targets to prevent or slow the development of ARHL.

## Abbreviations

ARHL: age-related hearing loss
CAS: central auditory system
CD: common deletion
D-gal: D-galactose
mtDNA: mitochondrial DNA
NOX: NADPH oxidase
PAS: peripheral auditory system
ROS: reactive oxygen species
T-AOC: total antioxidant capability
UCP: uncoupling protein
8-OHdG: 8-hydroxy-2-deoxyguanosine
